# Whole Genome and Transcriptome Sequencing of Two Multi-Drug Resistant *Mycobacterium tuberculosis* Strains to Facilitate Illustrating Their Virulence *in vivo*

**DOI:** 10.3389/fcimb.2020.00219

**Published:** 2020-05-15

**Authors:** Jun Tang, Zhihao Liu, Ya'nan Shi, Lingjun Zhan, Chuan Qin

**Affiliations:** ^1^Institute of Laboratory Animal Sciences, Chinese Academy of Medical Sciences (CAMS), Comparative Medicine Center, Peking Union Medical College (PUMC), Beijing, China; ^2^NHC Key Laboratory of Human Disease Comparative Medicine, Beijing, China; ^3^Beijing Key Laboratory for Animal Models of Emerging and Reemerging Infectious, Beijing, China; ^4^Beijing Engineering Research Center for Experimental Animal Models of Human Critical Diseases, Beijing, China; ^5^Tuberculosis Center, Chinese Academy of Medical Sciences (CAMS), Beijing, China

**Keywords:** whole genome re-sequencing, RNA-seq, transcriptome sequencing, *Mycobacterium tuberculosis*, multi-drug resistant, virulence

## Abstract

*Mycobacterium tuberculosis* clinical strains usually possess traits different from the laboratory strains like H37Rv, especially those clinical drug resistant strains. With whole genome and transcriptome sequencing, we depicted the feature of two multi-drug resistant Mtb strains in resistance and virulence. Compared with H37Rv, the differential expressed genes (DEGs) of the MDR strains showed featured enrichment in arginine biosynthesis, fatty acid biosynthesis, and metabolism pathway. In the subset of virulence genes, the overlapping DEGs of the MDR strains exhibited downregulation of the cluster in type VII secretion system. In the mice experiment, the MDR strains showed attenuated but distinct virulence, both in survival rate and pathology. Taken together, the whole genome and transcriptome analysis could help understand the unique feature of the MDR strains both in resistance and virulence.

## Introduction

The genotype of *Mycobacterium tuberculosis* is an important factor in the interaction between the bacilli and the host, as well as in the preclinical study of drugs and vaccines. There is wide difference in the host immune responses such as cytokine induction, and in the effect of therapeutics when confronting with different strains (Krishnan et al., 2011; Reiling et al., 2013). Therefore, clinical isolates including drug resistant strains, are often used in tuberculosis research, in addition to the laboratory strains such as H37Rv or Erdman. However, the selected clinical isolates may vary in strain lineage, and the genomic information may not be fully revealed, thus the comparison among these studies are difficult to conduct. Recently, rapid development of sequencing technologies such as genome re-sequencing and RNA Seq greatly facilitates the investigation in the genomic information of clinical strains, upgrades the phylogenic and drug resistance analysis, and promotes the characterization of strain-specific gene expression modes (Van den Bossche et al., 2019). Besides, the linkage establishment between genotype and phenotype has also been accelerated, as well as the comparison among studies. There are also attempts made to select representative sets of isolates covering all lineages with clear genomic information, to establish reference clinical strains of Mtb, and further to promote the research using clinical strains (Borrell et al., 2019).

Here we selected two multi-drug resistant clinical Mtb strains isolated in China, performed whole genome re-sequencing and RNA-Seq, analyzed the drug resistant variations, and compared their strain-specific virulence patterns in C57BL/6 mice, to provide a comprehensive description on the characteristics of each strain. Compared with H37Rv, the MDR strains showed featured transcriptional enrichment in arginine biosynthesis and fatty acid biosynthesis and metabolism pathway, as well as downregulation of virulence genes in secretion system. Phenotypically, they exhibited attenuated but distinct virulence in mice. The genomic and transcriptomic analysis provided useful information for the investigation and further application of these two MDR clinical strains.

## Materials and Methods

### Bacteria Strains and Culture

*Mycobacterium tuberculosis* H37Rv (ATCC), clinical isolated strains 8462, 94789 (from National Institutes for Food and Drug Control) experienced one passage *in vitro* in Middlebrook 7H9 liquid medium supplemented with 10% OADC at 37°C before transcriptome sequencing. The two stains were previously isolated from the sputum specimens of two domestic patients with pulmonary tuberculosis. The culture was ultrasonic dispersed, diluted and plated on Lowenstein-Jensen slopes. Colonies were numerated after 3–4 weeks culture to calculate the cfu.

### *In vitro* Growth and Drug Sensitivity Test

*In vitro* growth and DST was performed with Bactec MGIT 960 system according to the protocols of the manufacturer (Tang et al., 2017). Briefly, 10^3^CFU of H37Rv, strain 8462 or 94789 was inoculated into MGIT 7mL tubes with growth supplement and pollution-inhibiting PANTA, then the tubes were scanned into the MGIT 960 system to be cultured and under automated monitoring. The time between tuber entry to automatedly determined positive was defined as time-till-detection (TTD). After turning cultured positive, 0.5 mL of each culture was inoculated into MGIT 7 mL tubes with SIRE supplement and isoniazid, rifampicin or streptomycin, then the tubes were scanned into the system to be cultured and the respective drug sensitivity was determined and reported.

### Mycobacterium Whole Genome Re-sequencing

Genomic DNA of each strain was extracted following standard methods (Somerville et al., 2005), and sequenced on the Illumina HiSeq 2500 platform to generate single-end reads. Reads with adapter, reads with more than 10% uncertain bases, and reads with 50% Q20 bases were removed during quality control. Valid reads were aligned to H37Rv reference genome sequence using BWA (Li and Durbin, 2010). SNPs were identified and filtered using SAMtools (Li et al., 2009), and annotated using ANNOVAR (Wang et al., 2010).

### Whole Genome-Based Strain Lineage and Antibiotic Resistance Analysis

The strain lineage analysis based on whole genome sequencing was performed through TB profiler (Coll et al., 2015) and PhyResSe (Feuerriegel et al., 2015). The antibiotic resistance analysis was using PhyResSe (www.PhyResSe.org).

### Transcriptome Sequencing

Samples were collected in duplicate from liquid cultures of H37Rv, strain 8462 and 94789 at day 14. For RNA sequencing, rRNA was depleted from the total RNA. Then the libraries were prepared under strand specific construction, and the quality was checked with Agilent 2100 Bioanalyzer. Sequencing was performed on the Illumina HiSeq 4000 platform. Reads were mapped against H37Rv reference genome using Bowtie2 to produce BAM files. Gene expression values were analyzed base on RPKM (reads per kilobase per million) using HTSeq with Union model. Genes with PRKM>1 were further put into differential expression analysis using DESeq (Anders and Huber, 2010).

### Animal Experiments and Pathology

The animal experiment protocols were approved by the Institutional Animal Care and Use Committee of the Institute of Laboratory Animal Sciences, CAMS & PUMC (approved No. ZLJ17002). For the survival experiment, ten animals per group were held. The 6–8-week-old C57BL/6 female mice were infected with different strains of Mtb intravenously at a dosage of 2.5 × 10^7^CFU in 200 μ L of saline per animal, then the survival of the infected mice was daily monitored for 30 days. On daily surveillance, the infected mice which matched the condition of humane endpoint were euthanized by cervical dislocation in accordance with the approved protocols. To observe the tuberculosis lesions in organs, six female C57BL/6 mice per group were infected with different Mtb strains at 1 × 10^5^CFU/mouse intravenously, and were euthanized after 6 weeks. Lungs and spleens were collected and fixed in 4% formaldehyde for 72 h, followed by paraffin embedded sectioning and H&E stain. The micrographs were captured with NanoZoomer S60 (Hamamatsu). All the infected mice were maintained in the animal biosafety level 3 laboratory in ILAS.

### Statistical Analysis

*In vitro* experiments were repeated three times. One-way ANOVA was used for the statistical analysis to calculate *p* value.

### Data Availability

The acquired sequencing reads have been submitted, with the SRA accession PRJNA600079 in the NCBI Sequence Read Archive (SRA). The data are available at https://www.ncbi.nlm.nih.gov/Traces/study/?acc=PRJNA600079.

## Results

### Clinical Strains 8462 and 94789 Are Multi-Drug Resistant Mtb and Show Rapid Growth in Culture

**Figure 1:**
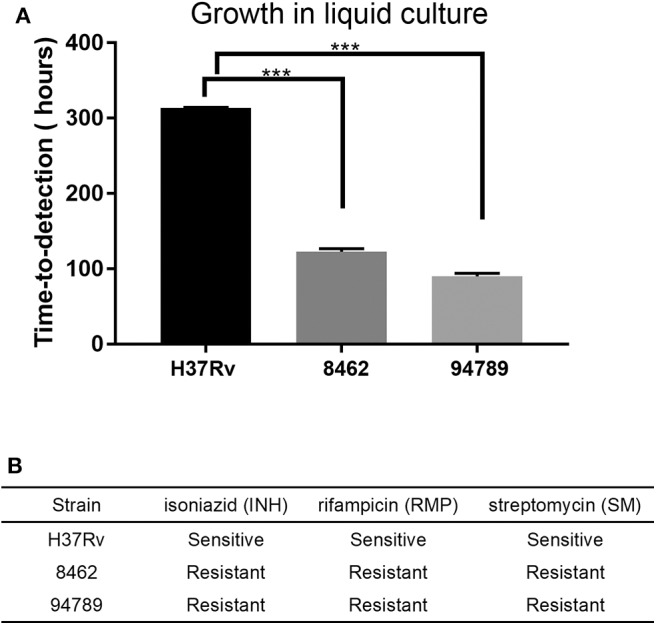
The growth and drug resistance of the Mtb strains in liquid culture. **(A)** The replication rate of each strain was assessed by time-to-detection in MGIT 960 system automated culture, from 10^3^CFU inoculation to report positive. *n* = 3, ***p < 0.001. **(B)** Drug resistance phenotypes were determined in MGIT 960 system with SIRE kit. INH, 0.1 μg/mL; RMP, 1 μg/mL; SM, 1 μg/mL.

Two clinically reported multi-drug resistant Mtb strains 8462 and 94789 were selected. The replication rates of these strains in liquid culture were determined in MGIT 960 system (Figure 1A). For strains 8462 and 94789, it cost much shorter time with a very low P value than H37Rv to turn culture positive from the initial inoculation of the same cfu. Their resistance to isoniazid, rifampicin and streptomycin was also verified in MGIT 960 automated detection system (Figure 1B). Both of the clinical strains were resistant to all three antibiotics.

### Genome Re-sequencing Revealed Different Features of the Two Multi-Drug Resistant Strains

**Figure 2:**
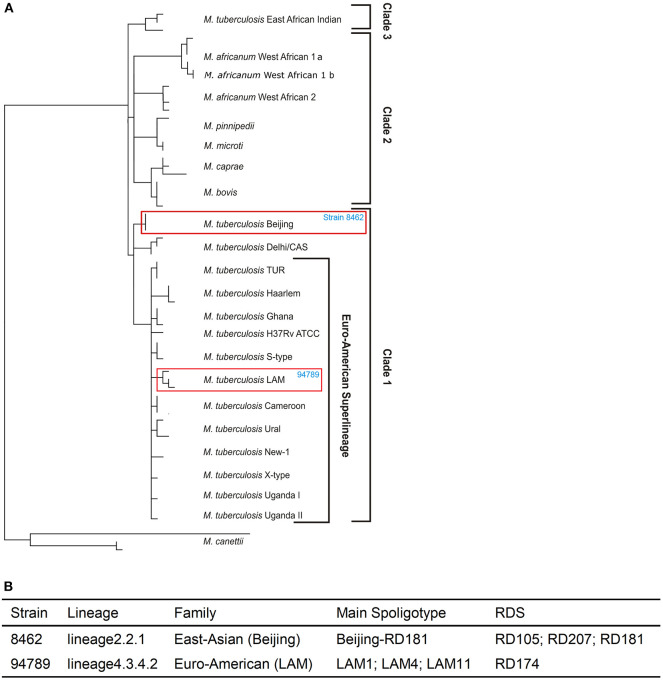
The two MDR strains are from different lineages. **(A)** The positions of strain 8462 and strain 94789 in the phylogenetic tree. **(B)** The detailed lineages of strain 8462 and strain 94789. Analysis using TB Profiler.

Whole genome re-sequencing was performed to identify and analyze the variants of strains 8462 and 94789. We identified 1954 variants in strain 8462, and 1145 variants in strain 94789. Phylogenic analysis based on SNP categorized strain 8462 into lineage2.2.1 of Beijing genotype, while strain 94789 into lineage4.3.4.2 of LAM genotype (Figures 2A,B and Supplementary Table 1). The corresponding Spoligotype and RDs were also listed.

We further analyzed the drug resistance of these two strains using PhyResSe, based on the whole genome sequencing data. Highly confident SNPs which had been confirmed in previous research were used to illustrate the separate resistance (Tables 1, 2). In strain 8462, variants causing resistance to fluoroquinolones, rifampicin, streptomycin and isoniazid were recognized, while in strain 94789 only rifampicin and isoniazid resistant variants were recognized.

**Table 1:** High confident drug resistant variants in strain 8462.

**Position**	**Mutation in genome direction**	**Gene name**	**Dir**.	**AA change**	**Codon change**	**Antibiotic**	**Reference PMID in PubMed**	**High confidence SNP**
7581	G → T	gyrA	+	Asp94Tyr	gac/tac	Fluoroquinolones (FQ)	21562102	Yes
761140	A → G	rpoB	+	His445Arg	cac/cgc	Rifampicin (RMP)	9003625	Yes
781687	A → G	rpsL	+	Lys43Arg	aag/agg	Streptomycin (SM)	22646308	Yes
2155168	C → G	katG	–	Ser315Thr	agc/acc	Isoniazid (INH)	8878604	Yes

**Table 2:** High confident drug resistant variants in strain 94789.

**Position**	**Mutation in genome direction**	**Gene name**	**Dir**.	**AA change**	**Codon change**	**Antibiotic**	**Reference PMID in PubMed**	**High Confidence SNP**
761155	C → T	rpoB	+	Ser450Leu	tcg/ttg	Rifampicin (RMP)	21300839	Yes
2155168	C → G	katG	–	Ser315Thr	agc/acc	Isoniazid (INH)	8878604	Yes

### RNA-seq Described Unique Transcriptional Features of the MDR Strains

**Figure 3:**
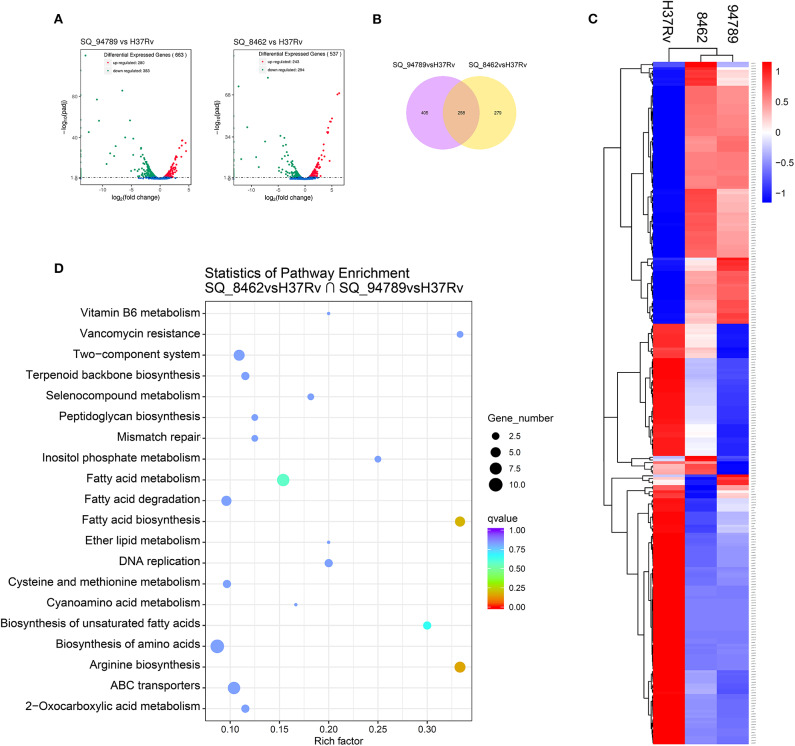
Analysis of the overlapping differential expressed genes of the MDR strains. **(A)** Volcano plot displaying differential expressed genes between strain 94789 and H37Rv (*n* = 663 genes), and DEGs between 8462 and H37Rv (*n* = 537 genes). x-axis: log2(fold change); y-axis: -log10(adjusted p-value). **(B)** Venn diagram represents the numbers of overlapping differential expressed genes of the two MDR strains relative to H37Rv. **(C)** Heatmap of the clustered overlapping DEGs (*n* = 258 genes) in **(B)**. **(D)** KEGG pathway enrichment of the overlapping DEGs in **(B)**.

We further performed RNA-seq on reference strain H37Rv and these two MDR strains from Middlebrook 7H9 culture at day 14. There are 663 differentially expressed genes (DEGs) between H37Rv and strain 8462, and 537 DEGs between H37Rv and strain 94789 (Figure 3A). Genes with fold change >2 or smaller than 0.5, and q value smaller than 0.05, were defined as DEGs. These two sets of DEGs were compared via Venn diagram tool and 258 overlapping genes were recognized (Figure 3B).

Then we performed cluster analysis on the subset of these overlapping DEGs (Figure 3C and Supplementary Table 2). There is relatively high similarity between strain 8462 and strain 94789 in the expression of this subset. These 258 overlapping DEGs were further significantly enriched into four KEGG pathways including arginine biosynthesis, fatty acid biosynthesis, fatty acid metabolism, and biosynthesis of unsaturated fatty acids (Figure 3D and Table 3).

**Table 3:** The enriched KEGG pathways of the overlapping DEGs.

**Term**	**KEGG PATHWAY ID**	**Input number**	**Background number**	***P*-Value**	**Input gene symbol**
Arginine biosynthesis	mtu00220	6	18	0.002463	argF, argH, argG, argJ, argD, argB
Fatty acid biosynthesis	mtu00061	5	15	0.005691	desA2, fadD15, desA1, fabG4, fabD2
Fatty acid metabolism	mtu01212	8	52	0.026593	desA1, fadE23, fadD15, desA2, echA9, fabD2, fadA2, fabG4
Biosynthesis of unsaturated fatty acids	mtu01040	3	10	0.039797	desA1, fabG4, desA2

### Transcriptomic Analysis of Virulence Factors Showed Low Transcription of Secretion System in the MDR Strains

**Figure 4:**
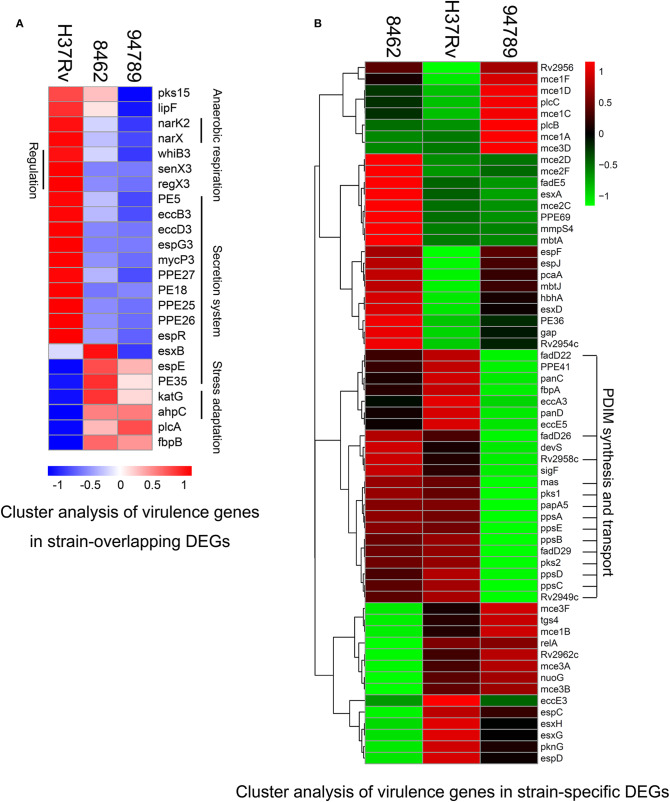
Cluster analysis of the differential expressed virulence genes in strain 8462, 94789 and H37Rv. **(A)** Heatmap displaying the virulence genes in overlapping DEGs of strain 8462 and 94789 (*n* = 24 genes). **(B)** Heatmap displaying the virulence genes in the strain specific DEGs of strain 8462 or 94789 (*n* = 61).

To observe the transcriptional pattern of the known virulence factors in the MDR strains, we listed and categorized all the genes known related to mtb virulence in the above 258 overlapping DEGs (Forrellad et al., 2013). There are 24 genes identified in several categories. Genes in phthiocerol dimycocerosate (PDIM) synthesis (pks15), lipase (lipF), anaerobic respiration (narK2, narX), transcriptional regulation [whiB3, senX3-regX3 (two component system)], type seven secretion system (PE5, eccB3, eccD3, espG3, mycP3, PPE27, PE18, PPE25, PPE26, espR) were at lower expression level in both of the MDR strains (Figure 4A). The decrease in mainly secretion system in the MDR strains may suggest a defensive mechanism of resistant bacilli. A small number of genes were at higher expression level in the MDR strains compared with H37Rv, including espE and PE35 (ESX-1 secretion), katG and ahpC (oxidative stress adaptation), plcA (lipid synthesis) and fbpB (secreted protein Ag85B). Distinctively, esxB, also known as cfp10, expressed higher in strain 8462 than in H37Rv, but lower in strain 94789 than in H37Rv (Supplementary Table 3).

We also identified the virulence genes carrying non-silent variants in both of the MDR strains in the whole genome re-sequencing data (Supplementary Table 4). On cross referencing, three virulence related DEGs (pks15, PE35, and katG) were found also carrying non-silent variants.

The strain specific differentially expressed virulence genes (61 genes) were also identified (Supplementary Table 5).

The cluster analysis demonstrated a featured downregulated cluster of PDIM synthesis and transport in strain 94789 but not in 8462 (Figure 4B). PDIMs are complex cell wall lipids which are closely related to mycobacteria virulence, implicating in the phagosomal escape and host cell exit of Mtb (Day et al., 2014; Quigley et al., 2017).

### Strain 8462 and 94789 Showed Attenuated Virulence in Mice

**Figure 5:**
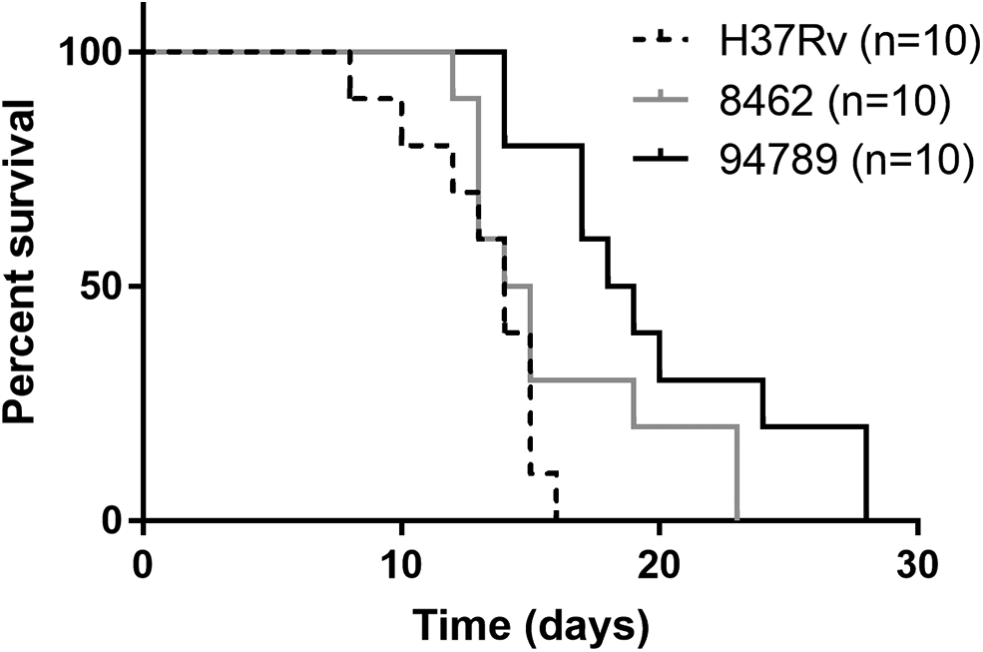
Mice survival after challenge of different Mtb strains. Kaplan-Meier survival curves for 2.5 × 10^7^CFU H37Rv, strain 8462 or 94789 intravenously challenged C57BL/6 mice (*n* = 10).

**Figure 6:**
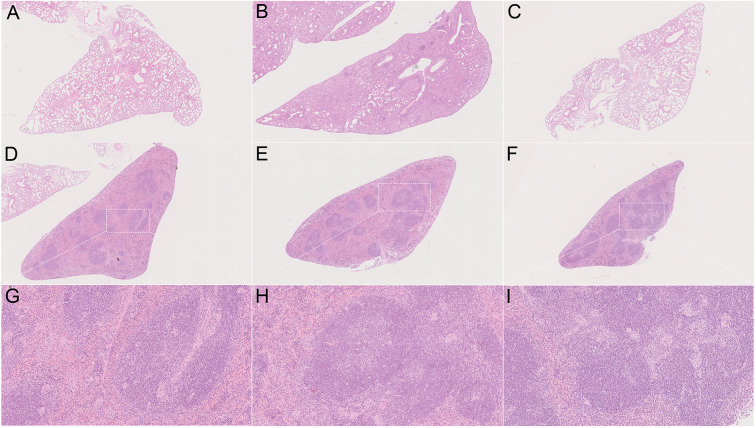
Pathology on challenge of different Mtb strains. Representative H&E stained sections demonstrate distinct lesions caused by H37Rv **(A,D,G)**, strain 8462 **(B,E,H)**, and 94789 **(C,F,I)** in lung **(A–C)** and spleen **(D–I)** of C57BL/6 mice after 6 weeks infection. 25 × **(A–F)** and 100 × **(G–I)** magnification.

In order to determine the *in vivo* virulence of these strains, we challenged C57BL/6 mice with H37Rv, strain 8462 or strain 94789 intravenously. On challenging with high dose (2.5 × 10^7^CFU) of Mtb, both groups of the MDR strains infected mice showed higher survival than H37Rv group within 30 days, and strain 94789 infected mice showed the highest survival (Figure 5). When challenged with low dose (1 × 10^5^CFU) for 6 weeks, the MDR strains infected mice developed pathological lesions in lungs and spleens distinct from those in the H37Rv infected mice (Figures 6A,D,G). There were severe diffuse infiltration and structure damage in the lungs of strain 8462 infected mice, but the spleens bear mild damage (Figures 6B,E,H). On the contrary, infection of strain 94789 caused slight pathological injury in lungs, while resulted in severe lesions in spleens comparable with those in H37Rv infected mice (Figures 6C,F,I).

## Discussions

In this study we described the unique features of two MDR-TB strains isolated from China both in phenotype and genotype, including the *in vitro* growth and drug resistance phenotype, whole genome and transcriptome sequencing, and survival and pathology in mice. Through these comprehensive analysis, we try to generate new information and clues on the linkage between the phenotype and the genotype. Whole genome sequencing has become a powerful tool of individualized treatment for TB patients. It has high sensitivity and specificity on drug resistance detection, and also on the pathogen transmission and evolution analysis, both important for the decision of therapeutic regimen. Here, the resistance to isoniazid and rifampicin were well-detected based on WGS in both of the MDR strains, confirmed by DST in MGIT 960 system. However, the resistance to streptomycin in strain 94789 was not predicted in WGS analysis as in strain 8462 because of lacking verified resistant variants. But we found additional non-silent mutations within the CDS of rrs and gidB, which may contribute to the streptomycin resistant phenotype (Supplementary Table 1).

The different transcriptional patterns between drug susceptible strains and drug resistant strains have been extensively investigated recently, as it is quite likely that the acquired mutations from selection could result in transcriptional changes (de Welzen et al., 2017). Downregulation of ethA, which was identified as a new mechanism of ethionamide resistance in XDR strains previously, was also observed both in MDR strains 8462 and 94789 here (Supplementary Table 2) (de Welzen et al., 2017). Increased expression of the efflux pump gene pstB, which may contribute to drug resistance in Mtb, was found in strains 8462 and 94789 as well (Supplementary Table 2) (Li et al., 2015). Thus, although strain 8462 was from the Beijing branch of lineage 2 and strain 94789 was from the LAM branch of lineage 4, they possessed considerable similarity in SNPs and much more in transcriptomic patterns as members of MDR Mtb.

The overlapping 258 DEGs in strains 8462 and 94789 were most enriched into arginine biosynthesis pathway, as well as fatty acid biosynthesis and metabolism pathway. A recent research highlighted the importance of the *de novo* arginine biosynthesis pathway for Mtb in the oxidative stress toleration (Tiwari et al., 2018). On treatment with isoniazid or vitamin C, four members (argB, argC, argD, argJ) of the pathway were upregulated to response (Tiwari et al., 2018). Accordingly, mutants ΔargB and ΔargF were rapidly cleared in unsupplemented media or in mice, suffering from accumulation of ROS and extensive DNA damage (Tiwari et al., 2018). Here, six of eight enzymes in the *de novo* arginine biosynthesis pathway were identified in the overlapping DEGs, making it the most significant transcriptomic characteristic of the two MDR strains.

In the mice experiments, both of the MDR strains exhibited attenuated and distinct *in vivo* virulence compared with H37Rv. This could be partially attributed to the negative effect of the drug-resistance-conferring variants on Mtb fitness and virulence, including variants in katG as reported (Nieto et al., 2016). As multi-drug resistant strains, strain 8462 and 94789 may have undertaken multiple drug selection pressure, therefore their virulence was impaired probably by multiple variants synergistically. We used the transcriptional patterns and the non-silent mutations of the virulence factors in these MDR strains to directly depict the possible virulence genes which could be involved in the attenuated virulence. A decreased transcription of a cluster of secretion system genes was found in both of the MDR strains, and further a cluster of PDIM synthesis and transport genes was found to decrease in strain 94789 only. It is noted that PDIM synthesis is usually under negative selection during *in vitro* culture (Domenech and Reed, 2009). Although we have avoided repeat subculture of the clinical strains, the transcriptional difference in the cluster of PDIM synthesis should be cautiously examined on their clinical relevance. Besides, an enzyme involved in PGL biosynthesis, pks15, which was proposed to be a marker for highly virulent strains, was downregulated in both strain 8462 and 94789 (Constant et al., 2002; Reed et al., 2004). However, H37Rv does not produce PGLs, as the deletion of a guanine causes a frameshift and loss function of Pks15 (Constant et al., 2002). This frameshift was also found in strain 94789, but in strain 8462 the frameshift was corrected by an insertion of seven nucleotides (CCGCGGC). As Pks15 from the correct ORF with two extra amino acids is reported functional in M. tuberculosis Canetti, pks15 could be considered as one of the factors under the distinct virulence of these three strains.

The MDR strains in this study showed rapid replication rate *in vitro*, which is an interesting feature when we selected the objects of the study. However, the resulting small sample size is one of our limitations. Besides, we have little information about the clinical founding of the patients from whom the MDR strains were isolated. The combining study of the clinical founding, multi-omics analysis, and animal experiment would provide more inspiration about the pathogenesis of the MDR Mtb.

Mining the sequencing data provided useful information for us to illustrate the resistance and the virulence of these MDR strains. Recently, transcriptome sequencing was more and more used to investigate the mechanisms of antibiotics and the response of different Mtb strains (Briffotaux et al., 2019; Van den Bossche et al., 2019). Following whole genome sequencing, RNA-seq is becoming another powerful tool for the research on tuberculosis.

## Data Availability Statement

The acquired sequencing reads have been submitted, with the SRA accession PRJNA600079 in the NCBI Sequence Read Archive (SRA), https://www.ncbi.nlm.nih.gov/Traces/study/?acc=PRJNA600079.

## Ethics Statement

The animal study was reviewed and approved by Institutional Animal Care and Use Committee of the Institute of Laboratory Animal Sciences, CAMS&PUMC.

## Author Contributions

JT acquired and analyzed the data and wrote and revised the manuscript. LZ and CQ designed and supervised the study and revised the manuscript. YS assisted in the mice experiment and pathology. ZL was responsible for the bacterial culture, drug sensitivity test, mice challenging, and pathology.

## Conflict of Interest

The authors declare that the research was conducted in the absence of any commercial or financial relationships that could be construed as a potential conflict of interest.
