# Supplementary material for: Whole Genome and Transcriptome Sequencing of Two Multi-Drug Resistant Mycobacterium tuberculosis Strains to Facilitate Illustrating Their Virulence in vivo
Source: Front Cell Infect Microbiol. 2020 May 15;10:219. doi: 10.3389/fcimb.2020.00219 (PMC7242654; doi:10.3389/fcimb.2020.00219)
Supplement: Supplementary file 4 [file Table_4.docx]

| Gene name | Gene ID | Type | Strain 8462 | Strain 94789 | Category |
| --- | --- | --- | --- | --- | --- |
| nuoG | Rv3151 | SNP | Ile474Met | Ile474Met | Anti-apoptosis factor |
| nrp | Rv0101 | SNP | Leu659Phe | Val2000Val | GPL locus |
| ppsA | Rv2931 | SNP | Asp624Glu | Asp624Glu | PDIM biosynthesis |
| pks15 | Rv2947c | SNP | INSERTION | Val333Ala | PGL biosynthesis |
| kefB | Rv3236c | SNP | Thr102Ala | Ala370Thr | Potassium/proton antiporter |
| mce1D | Rv0172 | SNP | Ile188Thr | Ala265Val | mce operons |
| mce1F | Rv0174 | SNP | Leu370Pro | Leu370Pro | mce operons |
| mce2A | Rv0589 | SNP | Phe51Ser | Phe51Ser | mce operons |
| mce3F | Rv1971 | SNP | Ala396Glu | Pro331Arg | mce operons |
| mbtH | Rv2377c | SNP | Val69Ala | Val69Ala | Mycobactin-Metal uptake |
| mbtF | Rv2379c | SNP | Glu589Asp | Glu589Asp | Mycobactin-Metal uptake |
| mbtB | Rv2383c | SNP | Val674Leu | Ser412Arg | Mycobactin-Metal uptake |
| mprB | Rv0982 | SNP | Leu339His | Ser498Ala | Regulation |
| phoR | Rv0758 | SNP | Gln59Pro | Pro172Leu | Regulation |
| sigM | Rv3911 | DEL | - | - | Regulation |
| PE35 | Rv3872 | SNP | Glu99STOP | Glu99STOP | Secretion system |
| espA | Rv3616c | SNP | Thr192Ile | Thr192Ile | Secretion system |
| Rv3888c | Rv3888c | SNP | Arg284Pro | Ile16Val | Secretion system |
| PPE69 | Rv3892c | SNP | Thr19Lys | Thr19Lys | Secretion system |
| eccC2 | Rv3894c | SNP | Asp650Gly | Arg258Pro | Secretion system |
| eccC3 | Rv0284 | SNP | Pro214Arg | Pro214Arg | Secretion system |
| eccE3 | Rv0292 | SNP | Asn217Asp | Asn217Asp | Secretion system |
| eccC4 | Rv3447c | SNP | Ser1082Gly | Ser1082Gly | Secretion system |
| katG | Rv1908c | SNP | Ser315Thr | Ser315Thr | Stress adaptation |

Supplementary Table 4. Virulence genes with non-silent variants in both of the MDR strains. (PDIM, phthiocerol dimycocerosate; PGL, phenolic glycolipid; mce, Mammalian cell entry)
